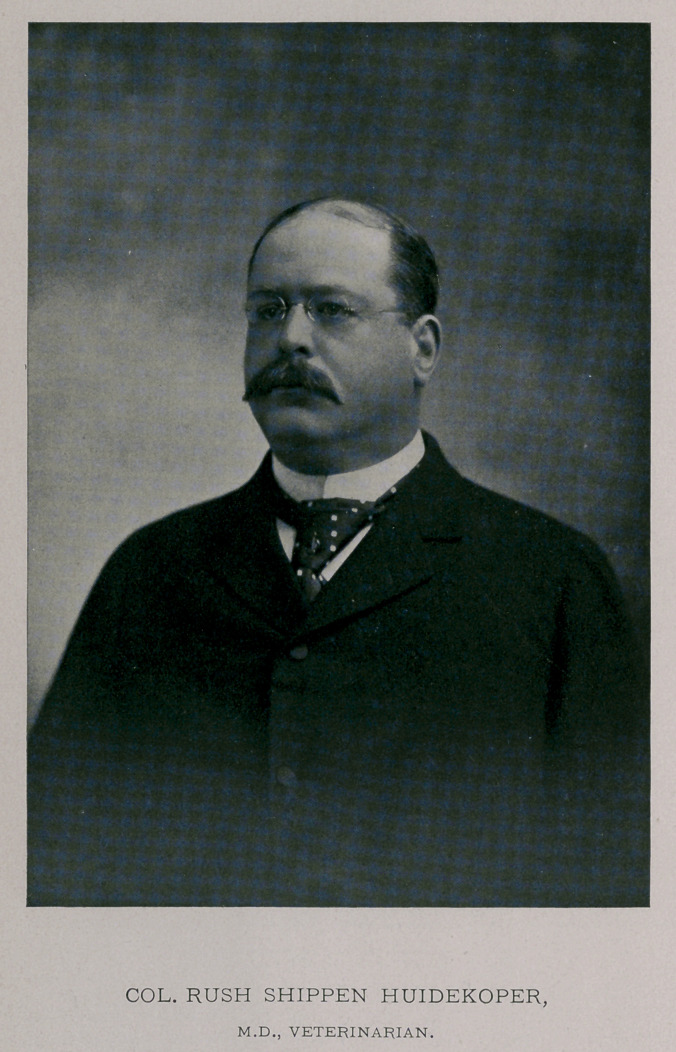# Rush Shippen Huidekoper—Physician, Surgeon, Veterinarian, Editor, Teacher, Author, Etc.

**Published:** 1901-12

**Authors:** 


THE JOURNAL

OF

COMPARATIVE MEDICINE AND VETERINARY ARCHIVES.

VOL. XXII. *DECEMBER, 1901.* No. 12.

### RUSH SHIPPEN HUIDEKOPER—PHYSICIAN, SURGEON, VETERINARIAN, EDITOR, TEACHER, AUTHOR, ETC.

BORN at Meadville, Pa., May 3, 1854, the son of Edgar Huidekoper, a well-known writer on theological subjects, and Frances Shippen, the daughter of Judge Henry Shippen. His grandfather was Harm Jan Huidekoper, the founder of the Meadville Theological Seminary.

Died at Philadelphia, Pa., December 17, 1901. Age, forty-seven years.

Dr. Huidekoper's death occurred at the Presbyterian Hospital, where he was taken for an operation to relieve a condition arising incident to an attack of pleurisy. Pneumonia followed the operation, from which he succumbed on the morning of the 17th of December, after an illness of ten weeks.

He was married March 15, 1877, to Anne Preston Morris, who survives him.

The burial services were held at Meadville, Pa., Thursday, December 19, 1901, and his body laid at rest amid the scenes of his childhood and in sight of the old homestead that he had ever held in tender memories.

His early education was obtained at Phillips Academy, Exeter, N. H. He was graduated from the University of Pennsylvania, Medical Department, in 1877; from the National Veterinary College of France, at Alfort, in 1882.

Several years of his life were spent in active practice and in teaching, and upon the staff of the Children's Hospital, Philadelphia Dispensary, and University Hospital, and for two years he served as Coroner's physician of the city of Philadelphia.

In completing his education abroad he devoted many months in the laboratories of Virchow, Koch, Chauveau, and Pasteur.

After spending several years abroad he returned in 1883 to undertake the work of founding a veterinary department of the University of Pennsylvania, with which department he remained until 1889, when he removed to New York, and for a time was among the corps of professors of the American Veterinary College, after which he accepted the Professorship of Anatomy and Surgery in the New York College of Veterinary Surgeons, and remained with that institution until the amalgamation of the two schools, at which time he was appointed by President McKinley as Chief Surgeon of the First Army Corps, stationed at Chickamauga, with the rank of Lieutenant-Colonel of Volunteers.

On closing his career with the Spanish-American War he returned to Washington, to take up for the American Veterinary Medical Association and the veterinary profession of the United States the battle for recognition, with rank, for the veterinarian in the United States Army, and with what courage and fidelity he fought this issue more than six thousand veterinarians of the United States speak only in the highest praise and remember with gratitude.

He was one of the first Board of Veterinary Medical Examiners in New York State, and gave valuable assistance in obtaining this legislation in the Empire State, and to his broad knowledge and unselfish devotion the general direction of this Board at its inception owes much of the strength and character of the good work they have done.

The National Horseshow of America, Open-air Horseshow, National Dogshow, National Bull Terriers' Club of America, all availed themselves of his broad education, true sportsman-like judgment, and training, and much of their success was largely contributed to by his efforts and labor. At New York, Philadelphia, Baltimore, and Toronto, Canada, he filled the rôle of judge and inspector with great satisfaction and assistance to their Horseshows and Live-stock Expositions.

His was a life of unceasing labor and effort in behalf of better conditions of every aspect of his profession's broadest work. An untiring worker and of ceaseless energy, which he applied to everything he selected to do, whether in teaching, in journalistic work, in writing, in army work of every description, in legislation, national, state, or municipal. Of unflinching courage and boundless enthusiasm in life, he exhibited a like fortitude and courage throughout all his illness, and with enthusiasm planned in his last sickness for another battle for recognition, with rank, for our profession in the United States Army.

Dr. Huidekoper was an honorary member of the Pennsylvania and Keystone Veterinary Medical Associations, member of the American Veterinary Medical Association, New York State Veterinary Medical Association, New York County Veterinary Medical Association, and a number of other medical and veterinary organizations, a member of the Philadelphia Club for many years, Rose Tree Hunt, Army and Navy Club of New York City, and many other social bodies and organizations for the development of true sport.

### Dr. Huidekoper's Work as a Teacher.

Dr. Huidekoper's older students look back upon the hours spent in his class-room as among the most profitable of their lives. And this is the true measure of a great teacher. When men of ripened judgment—men who have delved deeply into the mines of learning located and staked off by a master—find that he not only indicated truly the conditions they would find and the means of avoiding the difficulties they would encounter, but also implanted in them a desire to advance, a lust to discover, it is then evident that the teacher was no mere pedant, but that he possessed the knowledge of the scientist and the rare faculty of the true teacher.

The more Dr. Huidekoper's students learn in the school of life the more they value his instruction.

Dr. Huidekoper's first lessons in teaching were as a volunteer assistant to the renowned surgeon Dr. D. Hayes Agnew. He thus had an opportunity to acquire some of the faculty for teaching that helped to make his chief so famous. After a short period in this work, and a few years in general practice and in pathological investigation as Coroner's physician of Philadelphia, he was asked to go abroad to prepare himself to organize the teaching in the Veterinary School about to be established by the University of Pennsylvania. This he did in 1881, and returned in 1883 after having graduated from the National Veterinary School at Alfort, France, and after studying in Berlin and having visited most of the important veterinary schools in Europe.

Under his supervision the Veterinary School was built and the courses arranged. The course of instruction laid down by Dr. Huidekoper followed, as far as was possible within a three-year curriculum, the plan of instruction at Alfort, with such modifications as were made necessary by the means at his disposal and by special American conditions.

One leading idea was that the students should not only hear and see, but that they should *do*; that they should learn the handiwork of their profession as well as its principles. With this thought in mind, clinical instruction, operative surgery, and practical farriery were developed to a point before unapproached in America. Much laboratory work and dissecting were required, and the students were quizzed and reviewed as well as lectured to.

It is now most interesting to note that at this time the great schools of medicine are rapidly coming to the practical plan of instruction laid down by Dr. Huidekoper for his veterinary students seventeen years ago.

Dr. Huidekoper was a man of great versatility, of prodigious memory, and marvellous capacity for continuous effort. All of these were important factors in his work as a teacher.

During the first years of the school he taught anatomy, histology, the theory and practice of veterinary medicine, zoötechnics, and held clinics. Besides all of this he conducted a very large practice and edited this JOURNAL. Few men could have done one-half of the work that Dr. Huidekoper seemed to carry buoyantly.

Until the organization of the school was complete, two years after it opened, and the teaching was apportioned among an adequate corps of professors and instructors, Dr. Huidekoper worked habitually until after midnight; and was usually up at six o'clock in the morning, ready for an ice-water bath and another day of unremitting labor.

Dr. Huidekoper's lectures were marked less by brilliancy of delivery or beauty of diction than by lucidity, by methodical arrangement of matter, and by precision of statement. He spoke rapidly, but clearly, from copious notes, and always left in his hearers' minds a clear picture of the conditions described. His method was positive rather than argumentative, because he believed it was better for students to have presented to them the demonstrated facts and his deductions from them than the various conflicting views of the authorities. But in the greater controversial questions he was careful to state both sides.

By extensive reading he kept himself informed of all important work along veterinary lines, and always gave his classes the advantage of the most advanced teachings. His work was always "up to date."

Dr. Huidekoper was always ready to explain difficult or obscure points in his lectures, and was ready to give to his students as much time outside of the class-room as they needed or as his other numerous engagements would allow; but for a lazy or inattentive student he had no patience; for these so sharp were his questions and so biting his comments that they soon found his clinics and class-room most uncomfortable.

As a rule, Dr. Huidekoper's students were much attached to him personally. They were loyal to him and took great pride in his achievements and in the honors that came to him.

While he was not severe as a disciplinarian, he was strict and was fond of military order and punctuality. A studied reproof to a student came from him but rarely; when it came it was deserved, and it was couched in a form that caused it to penetrate deeply.

Unquestionably, Dr. Huidekoper was at his best as a teacher. His profound knowledge, his logical arrangement of ideas, his facility for drawing a clear word picture, and the mutual confidence that existed between him and his classes made it easy for him to impart instruction and a pleasure for his students to learn.

There is not one of Dr. Huidekoper's students who does not now have a sense of deep gratitude toward him, and who does not now feel a deep sense of personal loss and of profound sorrow.

LEONARD PEARSON, Dean,
Veterinary Department, University of Pennsylvania.

### Dr. Huidekoper as a Professional Man.

Replying to your favor containing such unwelcome news, I beg to say that I saw by the press dispatches of the 17th a report of the death of our friend, and wired you asking you to convey my sympathy to his family, and stating that the veterinary profession had lost its best friend. The first news I had of Huidekoper's illness was while in Chicago, attending the National Live-stock Meeting, when Pearson informed me that he was very ill. This made me extremely sad, on account of my personal friendship for him as well as having knowledge of the great blow his death would be to the veterinary profession at large. However, on returning home and receiving the JOURNAL, I saw that you believed in his convalescence and recovery, and this encouraged me to write him a letter, which was dated, I think, on the 15th, just two days before he died. The kind of man Huidekoper was only appears once in a century, and, unfortunately, they oftentimes have to die that their good deeds and qualities may be fully appreciated. I fear that many of the veterinarians of America have no appreciation whatever of Huidekoper's excellent qualities and of the great sacrifices he made unselfishly and wholly in the interests of the veterinary profession of America. You could write his epitaph in the few words that mean volumes : " Here lies a man."

On the day after his death I met Senator Carter on the street, and he said, " I see that poor Huidekoper is dead." I replied, " Yes, it is true." The Senator then said, " He was a prince of good fellows and the sort of a man the world can ill afford to lose." There is not a man in the profession to-day that can take his place or any part of it, I fear. Much that is good could be said about him, but when you had finished you would not have said enough. Peace be with him ! And may we all die deserving such praise as he is in justice entitled to.

Very truly yours,

M. E. Knowles,

State Veterinarian, Helena, Montana.

### Dr. Huidekoper as a State Board Examiner.

I was very much surprised and grieved to learn of the death of our esteemed friend and colleague, Dr. Rush Shippen Huidekoper. My mind was placed somewhat at ease by the Journal's report of his convalescence.

It would be almost impossible for me in words to estimate the value of his work as a member of the Empire State Board of Examiners. While it is true that a pen picture carries to our mind a vivid expression, yet it does not lie within my power at the present writing to draw such a picture as would do him justice or show his ability as an examiner. Not only was he ever ready in this capacity, but when our law was being passed in the Legislature he was like a sentinel on duty, ready to be called at any moment, and would respond in person, with his pen, or both, just as the case demanded.. While filling the position as an examiner, the Board, recognizing his superior training, elected him the first " question editor." As one learned man in the profession has said, " Dr. Huidekoper was an ideal examiner." These words, I think, express the opinion of any unbiased mind that knew him personally. After his removal to your State he remained willing to render our Board whatever assistance he could. At the meeting of our State Veterinary Society in September, 1901, the question arose as to whether or not we should accept Dr. Huidekoper's dues, as he was not a resident of this State. It was put to a vote of the Society, and, if my memory does not fail me, the voice of the members was unanimous to accept his dues, as we desired his membership. Let me assure you that I personally feel greatly benefited by my association with him.

WILLIAM HENRY KELLY,
Secretary of the New York State Veterinary Board of Examiners.

### AS A DEVOTED MEMBER OF HIS PROFESSION.

The profession at large will certainly mourn the loss of this most eminent coworker in the field of veterinary science, and particularly in the efforts put forth to secure adequate army veterinary legislation. The profession in America is greatly indebted to the arduous and earnest labors of Dr. Huidekoper to place veterinary training upon a higher plane. Would that we had many more as capable men, willing to devote their energies in this direction! The sympathy of the Kansas City Veterinary College goes to the widow of Dr. Huidekoper.

S. STEWART, V.S.,
Secretary of the American Veterinary Medical Association.

I shall mourn the loss as a good and sincere friend, and sympathize with you in losing your partner and coworker of the veterinary journalism and the advancement of the profession.

AUSTIN PETERS.

## Figures and Tables

**Figure f1:**